# Nitrogenous Nutrients Promote the Growth and Toxicity of *Dinophysis acuminata* during Estuarine Bloom Events

**DOI:** 10.1371/journal.pone.0124148

**Published:** 2015-04-20

**Authors:** Theresa K. Hattenrath-Lehmann, Maria A. Marcoval, Heidi Mittlesdorf, Jennifer A. Goleski, Zhihong Wang, Bennie Haynes, Steve L. Morton, Christopher J. Gobler

**Affiliations:** 1 Stony Brook University, School of Marine and Atmospheric Sciences, Southampton, New York, United States of America; 2 NOAA-National Ocean Service, Marine Biotoxins Program, Charleston, South Carolina, United States of America; 3 Estación Costera “J.J. Nágera”, Departamento de Ciencias Marinas (FCEyN), Universidad Nacional de Mar del Plata, Mar del Plata; & Consejo Nacional de Investigaciones Científicas y Técnicas (CONICET), Argentina; University of Connecticut, UNITED STATES

## Abstract

Diarrhetic Shellfish Poisoning (DSP) is a globally significant human health syndrome most commonly caused by dinoflagellates within the genus *Dinophysis*. While blooms of harmful algae have frequently been linked to excessive nutrient loading, *Dinophysis* is a mixotrophic alga whose growth is typically associated with prey availability. Consequently, field studies of *Dinophysis* and nutrients have been rare. Here, the temporal dynamics of *Dinophysis acuminata* blooms, DSP toxins, and nutrients (nitrate, ammonium, phosphate, silicate, organic compounds) were examined over four years within two New York estuaries (Meetinghouse Creek and Northport Bay). Further, changes in the abundance and toxicity of *D*. *acuminata* were assessed during a series of nutrient amendment experiments performed over a three year period. During the study, *Dinophysis acuminata* blooms exceeding one million cells L-1 were observed in both estuaries. Highly significant (*p*<0.001) forward stepwise multivariate regression models of ecosystem observations demonstrated that *D*. *acuminata* abundances were positively dependent on multiple environmental parameters including ammonium (*p* = 0.007) while cellular toxin content was positively dependent on ammonium (*p* = 0.002) but negatively dependent on nitrate (*p*<0.001). Nitrogen- (N) and phosphorus- (P) containing inorganic and organic nutrients significantly enhanced *D*. *acuminata* densities in nearly all (13 of 14) experiments performed. Ammonium significantly increased cell densities in 10 of 11 experiments, while glutamine significantly enhanced cellular DSP content in 4 of 5 experiments examining this compound. Nutrients may have directly or indirectly enhanced *D*. *acuminata* abundances as densities of this mixotroph during experiments were significantly correlated with multiple members of the planktonic community (phytoflagellates and *Mesodinium*). Collectively, this study demonstrates that nutrient loading and more specifically N-loading promotes the growth and toxicity of *D*. *acuminata* populations in coastal zones.

## Introduction

The spatial and temporal expansion and increased intensity of HABs is a globally recognized phenomenon [1, 2]. Marine HABs associated with human health syndromes, for example, paralytic shellfish poisoning (PSP), amnesic shellfish poisoning (ASP) and diarrhetic shellfish poisoning (DSP), are a growing human health and economic concern in many coastal regions [3–5]. These HABs are often associated with substantial economic losses due to the closure of toxic shellfish beds [6–8]. Given the human health threats that these toxin-producing blooms pose and the global increase in these events [1, 9] more research is needed to understand what promotes and sustains these blooms.

Anthropogenic loading of nutrients and organic matter are known to play a central role in the outbreak of multiple HABs [1–3, 10, 11]. Several studies have demonstrated that inorganic nitrogen (ammonium, nitrate) promotes the growth of various HABs [3, 11–13]. Similarly, multiple culture and field investigations have reported the stimulation of several HAB species by different dissolved organic substrates including urea, amino acids, peptides, humic acids, B-vitamins and uncharacterized organic substances [2, 3, 11, 14–18]. Such mixotrophic tendencies of harmful algae are not surprising given that the evolution of pathways to incorporate N from other sources beyond DIN would afford a competitive advantage over other phytoplankton when inorganic sources are depleted [3, 19]. Moreover, phagotrophy combined with phototrophy has been shown to significantly increase the growth rates of several HABs in comparison to strict autotrophic growth [19–21]. While the effects of both inorganic and organic nutrients on the growth and toxicity of multiple HAB species have been assessed, these effects are not fully understood for the mixotrophic dinoflagellate, *Dinophysis acuminata*.

Diarrhetic Shellfish Poisoning (DSP) is a globally significant human health syndrome most commonly caused by dinoflagellates of the genus *Dinophysis* [1, 9, 22] with few cases associated with the benthic dinoflagellate *Prorocentrum lima* [23, 24]. *Dinophysis* spp. synthesize okadaic acid (OA) and dinophysistoxins (DTXs), the causative toxins of DSP, as well as the pectenotoxins (PTXs) [25, 26] which are not associated with DSP but may be hepatotoxic and may promote the formation of tumors in mammals [25, 27]. While DSP is common in regions of Europe, South America and Asia [1, 9, 22], prior to 2008 North America had experienced few *Dinophysis*-related closures with all but one of these events occurring in Canada [28–31]. In recent years, however, North America has witnessed an expansion of *Dinophysis* blooms yielding shellfish containing DSP toxins exceeding the USFDA action level (160 ng g^-1^ of shellfish tissue) on the east (NY) [32], west (WA) [33] and Gulf coasts (TX) [34–36] of the United States. Interestingly, *Dinophysis* spp. have been historically found in these regions [33, 37–39] prior to the incidences of shellfish toxicity, suggesting these blooms may have become more intense and/or toxic in recent years. Understanding the factors promoting such phase shifts merit further exploration.

While the nutritional ecology of most harmful algae has been explored at length, there has been a dearth of such research on *Dinophysis* [40]. Only within the past decade have cultures of *Dinophysis* been established after positively identifying the food source of this obligate mixotroph, the ciliate *Mesodinium* (= *Myrionecta*), and establishing a three-step culturing system involving cryptophytes [41]. Therefore, nearly all information known regarding *Dinophysis* and nutrients has been gleaned from field observations, which have come to conflicting conclusions. Some correlative field studies have found no relationship between *Dinophysis* densities and nutrient concentrations [6, 42, 43]. A study in South Africa using ^15^N-labeled compounds found that *D*. *acuminata*–dominated communities had a high affinity for recycled N (ammonium), a trait that may provide a competitive advantage in nitrate depleted waters [44]. Additionally, the only study investigating the effects of nutrients on the production of okadaic acid and its derivatives in *Dinophysis* found contrasting results for the two *Dinophysis* species investigated [45]. Notably, the most recent and comprehensive review of *Dinophysis* ecology focused solely on phagotrophy as a prime nutritional mode for this alga [22] and thus far there is no evidence on the ability of *Dinophysis* to directly use nutrients [40]. Given the limited and conflicting nature of results obtained to date, more research is needed to understand the effects of nutrients on the growth and toxicity of *Dinophysis*.

This multi-year (2008–2012) field investigation assessed the dynamics of *Dinophysis acuminata*, its associated toxins, okadaic acid (OA), dinophysistoxins-1 (DTX1) and pectenotoxins (PTXs), and nutrients in two eutrophic estuaries. The effects of nutrients (organic and inorganic, nitrogen- and phosphorus-containing) on the abundance and toxicity of *Dinophysis acuminata* were examined by conducting nutrient amendment experiments using natural plankton communities from these two systems. In addition, forward stepwise regression analyses were performed to relate *Dinophysis* and its toxins to multiple environmental parameters while correlation matrices were used to relate *Dinophysis* to other members of the phytoplankton community during experiments.

## Materials and Methods

### Field sampling

**Fig 1:**
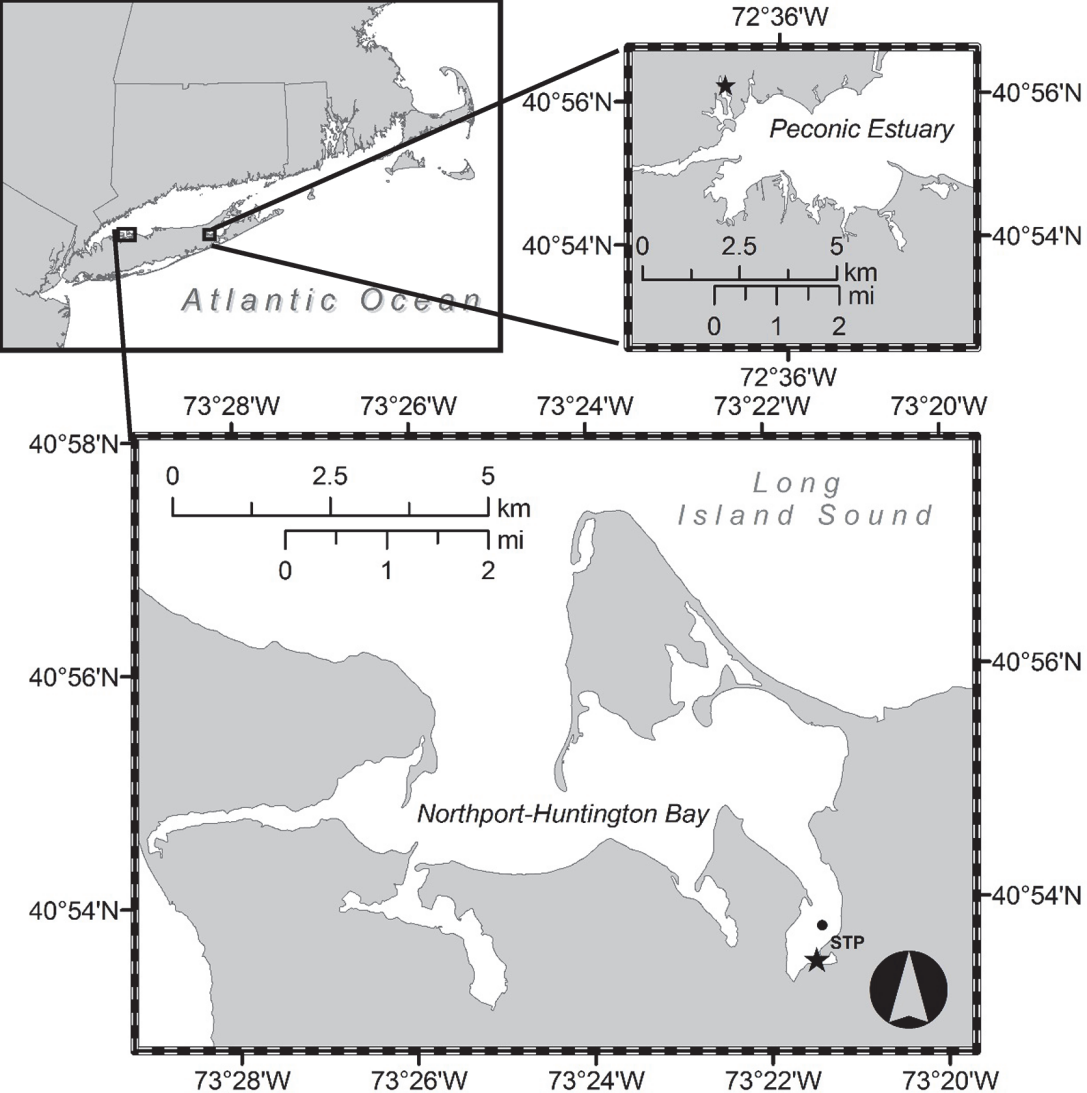
Field sampling (star) and sewage treatment plant outflow (circle) locations in the Northport-Huntington Bay complex and Meetinghouse Creek, New York, USA.

Field samples were collected on a weekly to twice-weekly basis from March through September during 2008 through 2012. Samples were collected in Northport Harbor (40°53.500′N, 73°21.434′W; Fig 1), a shallow (2 – 4m) well mixed system within the southeastern portion of the Northport-Huntington Bay complex, located on the north shore of Long Island, NY, USA [32]. Field samples were also collected weekly from March through August 2011 and 2012 from a second site in NY, Meetinghouse Creek, a tidal tributary located in the Peconic Estuary (40°56.314′N, 72°37.119′W; Fig 1). At each station, a YSI© 556 probe was used to record surface temperature, salinity and dissolved oxygen. Water was collected with permission by Peter Houmere from the Britannia Marina in Northport, NY. Further permission was not needed since seawater collection is not regulated by any municipal government agency.

Subsurface water (~0.25m) was filtered (in duplicate) for nutrient analysis using precombusted (4 hr @ 450^°^C) glass fiber filters (GF/F, 0.7 μm pore size) and frozen in acid washed plastic scintillation vials. Filtrate was analyzed colorimetrically for nitrate + nitrite, orthophosphate, ammonium and silicate [46, 47]. Total dissolved nitrogen and phosphorus (TDN, TDP) were analyzed by persulfate oxidation [48] and dissolved organic nitrogen and phosphorus (DON and DOP) levels were calculated by subtracting concentrations of nitrate + nitrite and ammonium or orthophosphate from concentrations of TDN and TDP, respectively. These analyses provided complete recovery of nitrate + nitrite, orthophosphate, ammonium, TDN, and TDP from Environmental Resources Associates (ERA, Golden, CO) certified reference material. For the determination of chlorophyll *a*, water was filtered in triplicate using glass fiber filters (GF/F; nominal pore size 0.7 μm) and measured using standard fluorometric techniques described in Welschmeyer [49]. Water samples were preserved in acidic Lugol’s solution at a final concentration of 2% (v/v). *Dinophysis* cell densities were enumerated using a 1mL Sedgewick-Rafter slide under a compound microscope using whole water samples and concentrated preserved water samples (*n* = 1 for 2008–2011, *n* = 2 for 2012). Prior to 2010 whole water samples were used for enumeration; however post-2010 concentrates were made to decrease the limit of detection as *Dinophysis* cell densities were often a relatively small portion of the total phytoplankton community and are therefore expressed as cells per L. Concentrated water samples were made by sieving 1–2L of water through either a 200 μm or 64 μm mesh (to eliminate large zooplankton) and then onto a 20 μm sieve that was backwashed into a 15 mL centrifuge tube. Counts made on plankton concentrates were not significantly different from direct counts on whole water. Detection limits for whole water and concentrated samples were 1,000 cells L^−1^ and 7 cells L^−1^, respectively.

Meteorological data including temperature, precipitation, wind direction and wind intensity were obtained for the months of April, May and June from the National Weather Service’s monitoring station in Islip, NY, USA which is ~20km from Northport. The 2008 bloom year was not considered in statistical analyses as the sampling resolution during June of that year was not equivalent to that of 2010, 2011 and 2012.

### Analysis of toxins in phytoplankton concentrates

Several liters of seawater were pre-sieved through a 200 μm mesh (to eliminate large zooplankton) and subsequently concentrated on a 20 μm sieve and backwashed into 15ml centrifuge tubes. Samples were centrifuged at 3000 rpm for 11 minutes and the supernatant aspirated without disturbing the cell pellet. Cell pellets were kept frozen at −20°C until further analysis. Algal pellets were resuspended in a known volume of 100% aqueous methanol, homogenized by vortex mixing and probe-sonicated (Branson 1450 sonicator) on ice at 30% amplitude, followed by centrifugation at 3400 × g for 10 min. The methanolic supernatants were filtered with a 0.2 μm syringe filter in preparation for analysis. Samples were analyzed for the presence of DSP toxins and the co-eluted pectenotoxins using liquid chromatography (HP 1100 series HPLC; Agilent Technologies, Palo Alto, CA) coupled with tandem mass spectrometry (4000 QTRAP hybrid triple quadrupole/linear ion trap mass spectrometer; AB Sciex, Foster City, CA) using the method described by Gerssen, Mulder [50] with modifications. LC separation was performed on a X-Bridge C18 (150 × 3 mm, 5 μm) column (Waters, Milford, MA) using a mobile phase of water (A) and acetonitrile/water (90:10, V/V) (B), both containing 6.7 mM ammonium hydroxide under gradient elution at a flow rate of 0.4 mL min^−1^ (linear gradient from 1min of 10% B to 90% B at 12 min, hold for 3 min, then return to 10% B at 17 min and hold for 4 min). The detection of DSP toxins by MS was achieved by multiple reaction monitoring (MRM) in negative ion mode for OA, DTX1, and DTX2 (for OA and DTX2 with MRM transitions of *m/z* 803.5 → 113.1and 255.1, for DTX1 with MRM transitions of *m/z* 817.5 → 113.1 and 255.1), and in positive ion mode for PTX2 and its isomers (MRM transitions of *m/z* 876.5 → 213.1 and 823.5), and PTX11 and its isomers (MRM transitions of *m/z* 892.5 → 213.1 and 839.5). Certified standards of OA, DTX1, DTX2, and PTX2 were available for toxin determination from NRC (Halifax, Canada) and RIKILT (Institute of Food Safety, The Netherlands). No standards were available for PTX11 and its isomers and PTX2 isomers; their concentrations were calculated approximately using PTX2 standards. PTX11 and its isomers showed different LC retention times and similar product ion spectra but differences at fragments between *m/z* 250 to 350 and some isomers could not match any of the published product ion spectra in this small mass range [51, 52]. PTX2 and its isomers also showed identical product ion spectra but different LC retention time. As such, all PTX concentrations were combined and reported as total PTXs (herein referred to as PTX). The detection limit was about 0.5 pg of OA, 0.65 pg of DTX1, 0.4 pg of DTX2, and 0.25 pg of PTX2 on LC column.

The toxin samples presented herein were not subjected to alkaline hydrolysis and therefore represent free acids (i.e. esterified toxins are not included) and are therefore lower than the total OA [32, 34]. This study did not quantify extracellular toxins which can account for a substantial fraction of the total DSP toxin pool [53–55]; the present study was well underway prior to the discovery of the importance of these extracellular toxins from *Dinophysis* and hence they were not considered here.

### Nutrient amendment experiments

To assess the impact of organic matter and nutrient loading on the abundance and toxicity of *Dinophysis acuminata*, a series of nutrient amendment experiments (dates initiated in parentheses) were performed during 2008 (12-May, 19-May and 26-May), 2010 (14-June, 22-June and 28-June) and 2011 (6-June, 13-June, 21-June, 27-June and 6-July). Triplicate bottles (2.5 L) were filled with water from Northport Harbor. While grazers were not removed from the water used to fill experimental bottles, stock water was kept well-mixed to ensure that grazers and phytoplankton were distributed evenly among all bottles. An unamended control was established along with several treatments (not all treatments were added on all experimental dates) including 20 μM nitrate, 20 μM ammonium, 10 μM urea (= 20 μM N), 10 μM glutamine (= 20 μM N), 2 μM phosphate, 100pM vitamin B_12_, 20 μM ammonium + 100pM vitamin B_12,_ and ~20 μM DON equivalent of high molecular weight organic matter from sewage treatment plant effluent (HMW STP) which also contained 5 μM ammonium. High molecular weight (>1 kDa, Millipore) organic matter from sewage treatment plant effluent was isolated and concentrated from the Riverhead Sewer District plant which is located in Riverhead, NY, ~50 km east of Northport and ~2 km west of Meetinghouse Creek. High molecular weight organic matter was isolated via tangential flow filtration as described by Gobler and Sanudo-Wilhelmy [56]. The use of tangential flow filtration ensures that high molecular weight organic material is concentrated while concentrations of low molecular weight compounds including inorganic nutrients remained unchanged [56]. Additional experiments were performed using bloom water from a second New York tributary, Meetinghouse Creek, during 2011 (6-April, 16-April and 9-May). Triplicate bottles (1 L) were filled with water from Meetinghouse Creek and an unamended control was established along with the treatments 100pM vitamin B_12_ and 100nM vitamin B_1_. All treatment concentrations were within the range of levels found in Long Island estuaries [57, 58], including Northport Bay [12], with the exception of glutamine which is typically present at submicromolar concentrations [59] but was added at equimolar levels of N for comparison with other nitrogen sources and vitamin B_1_ which can be required in large amounts by algal cultures [58]. Experiments did not involve any direct measurements of N or P incorporation. All bottles (2.5 or 1 L polycarbonate bottles) were incubated without mixing for ~48 h in an incubation chamber where light (photo period = 14:10 light:dark cycle) and temperature conditions where adjusted to match those found at the study site for each individual experiment. At the end of the 48 h incubation, toxin samples and size fractionated chlorophyll *a* (whole and >20 μm, chlorophyll samples were available only for Northport Bay experiments) were collected and analyzed as described above. *Dinophysis* cells and other members of the phytoplankton community were enumerated from samples preserved in Lugol’s solution. Plankton were identified and enumerated using a 1 mL Sedgewick-Rafter slide under a compound microscope. Cells larger than 10 μm were grouped as dinoflagellates, flagellates, diatoms (pennate vs. centric) and ciliates, and identified to genus level when possible. Phytoflagellates were generally too small (<10 μm) to identify and therefore some of the groups of flagellates, such as cryptophytes, that are potential indirect prey items for *Dinophysis* [40] were indistinguishable from other flagellates. Only *Dinophysis* densities were enumerated for Meetinghouse Creek experiments.

### Statistical analyses

For all experiments, differences among treatments were assessed via a one- way analysis of variance (ANOVA) using SigmaStat within SigmaPlot 11.0; when data sets failed normality tests, Kruskal-Wallis ANOVAs by ranks were performed. The Student-Newman-Keuls method was used as a post hoc, pairwise, multiple comparison procedures. A one-way ANOVA was used for interannual comparisons of nutrient concentrations, nutrient ratios, and meteorological data between years or seasons. Additionally, Spearman’s rank order correlation matrices were used to assess the extent to which *Dinophysis* densities co-varied with other members of the plankton community during experiments. Lastly, multiple regression analyses were performed to determine the dependence of *Dinophysis acuminata* abundances and toxicity on all measured environmental variables (i.e. inorganic nutrients, organic nutrients, plankton groups, temperature, salinity, chlorophyll *a*, etc) using forward stepwise selection (F to enter p < 0.049; F to remove p > 0.050) in SigmaStat within SigmaPlot 11.0. Cell densities were log transformed prior to analysis. The small bloom (Table 1) that occurred in 2009 during which okadaic acid or pectenotoxins were not detected [32] was not considered in statistical analyses.

**Table 1:** Peak *Dinophysis acuminata* densities and toxin concentrations in two New York estuaries, Northport Bay and Meetinghouse Creek.

Location	Year	*Dinophysis acuminata* (cells L^−1^)	Free OA (pg mL^−1^)	Free DTX1 (pg mL^−1^)	Total PTX (pg mL^−1^)
Northport Bay	2008	39,500	0.5	1.25	36.1
	2009	12,000	nd	nd	nd
	2010	116,000	2.0	8.75	105.2
	2011	1,266,000	4.2	20.4	2877.6
	2012	123,000	4.6	18.0	1112.7
Meetinghouse Creek	2011	38,000			
	2012	2,123,000	19.6	160.8	6764.8

n.d. = not detected.

## Results

### Dynamics of *Dinophysis*, toxins, nutrients and meteorological conditions in New York estuaries

**Fig 2:**
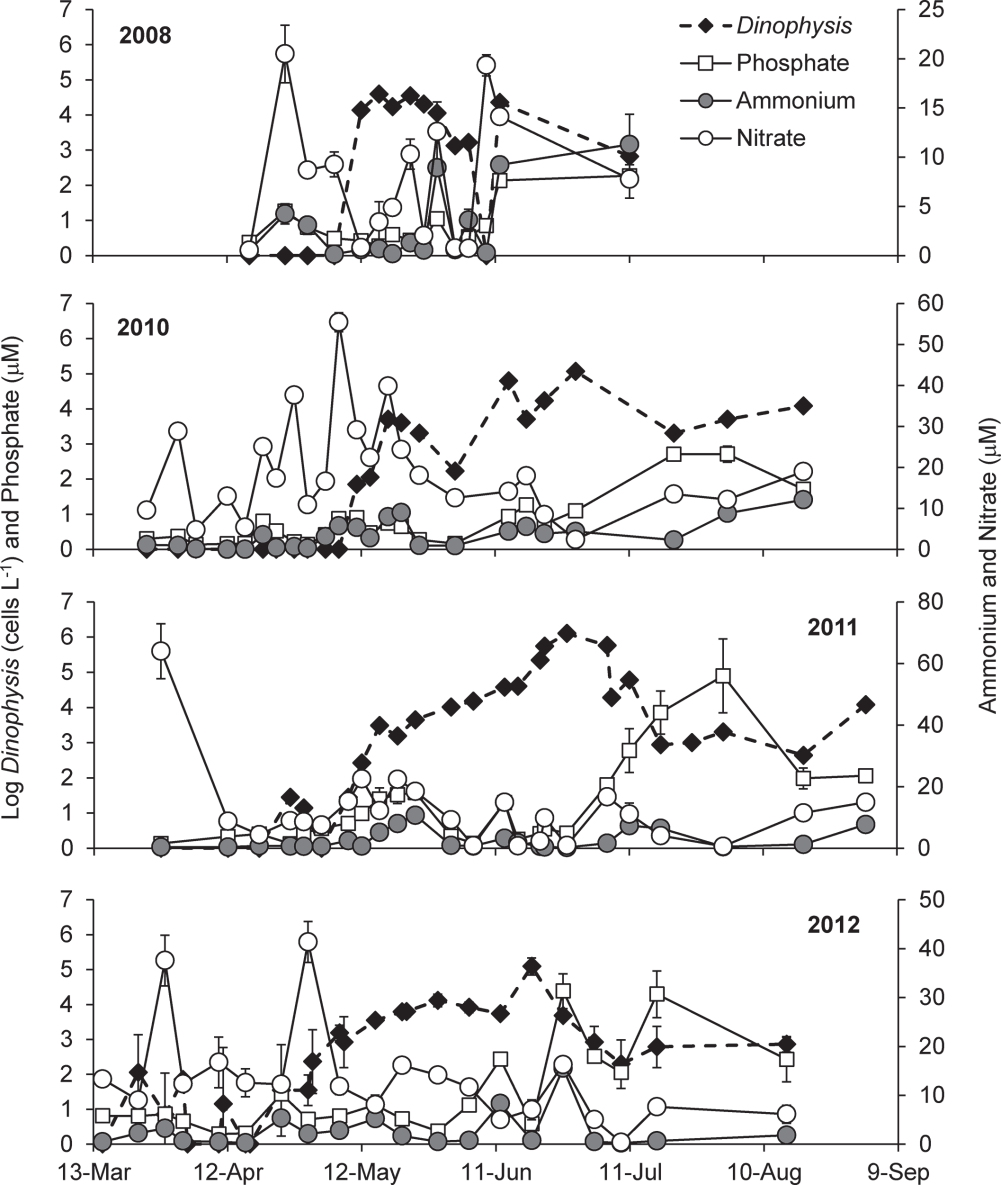
Log *Dinophysis acuminata* densities (cells L^-1^), and the inorganic nutrients, ammonium, nitrate and phosphate (μM) in Northport Bay, NY, USA during 2008–2012. Points are means while error bars represent the SD of duplicate samples.

Dense *Dinophysis acuminata* blooms were observed in Meetinghouse Creek and Northport Bay, with peak densities typically occurring between late April and June, at temperatures of 13–16°C and 19–24°C (13–17°C in 2008), for each respective estuary. When *D*. *acuminata* was present in the water column, temperature and surface salinities ranged from 8.7 to 26.6°C (mean ± SD = 18.9 ± 4.6°C) and 17.7 to 26.0 (mean ± SD = 24.0 ± 1) for Northport Bay, and 10.6 to 26.7°C (mean ± SD = 16.5 ± 5.2°C) and 8.2 to 26.7 (mean ± SD = 21.7 ± 5.3) for Meetinghouse Creek. Water column temperatures in Northport Bay were not statistically (*p* = 0.10) different among bloom years. In Northport Bay, the largest *D*. *acuminata* bloom occurred in 2011 reaching ~1.3 million cells L^−1^, followed by 2012, 2010, 2008, and 2009, with maximal densities of 123,000, 116,000, 39,500 and 12,000 cells L^−1^, respectively (Table 1 and Fig 2). Paralleling *D*. *acuminata* densities, maximal particulate toxin concentrations in Northport Bay occurred in 2011 with free OA = 4.2 pg mL^−1^, free DTX1 = 20.4 pg mL^−1^, and total PTX = 2,878 pg mL^−1^, while toxins were not detected in 2009 (Table 1). In 2012, *D*. *acuminata* densities in Meetinghouse Creek reached ~2.1 million cells L^−1^ (Table 1) during a bloom that persisted for ~2 months. Similar to Northport Bay, PTX was the most abundant particulate toxin measured in Meetinghouse Creek followed by free DTX1 and free OA, with concentrations of 6,770, 161, and 19.6 pg mL^−1^, respectively (Table 1). While there are multiple *Prorocentrum* spp. that often co-occur with *Dinophysis*: 1) none of these species are known DSP producers and 2) toxins were never detected when *Prorocentrum* was present and *Dinophysis* was absent.

Overall, mean chlorophyll *a*, and nutrient concentrations and ratios in Northport Bay varied interannually but were not significantly different among years (Table 2). Over the course of the entire study, mean concentrations of chlorophyll *a*, ammonium, nitrate, phosphate and silicate were 13.5 ± 2.6 μg L^−1^, 3.42 ± 0.46, 12.1 ± 1.13, 1.24 ± 0.14, and 33.7 ± 2.22 μM, respectively (Table 2). Dissolved organic N and P (DON, DOP) concentrations were 20.2 ± 1.10 and 0.80 ± 0.07 μM, while total dissolved N and P (TDN, TDP) were 35.7 ± 1.48 and 2.03 ± 0.16 μM, respectively (Table 2). Mean DIN:DIP, DON:DOP and TDN:TDP ratios were 20.6 ± 2.50, 35.3 ± 3.85 and 22.9 ± 1.73, respectively (Table 2). Finally, DIN:Si, DON:Si and TDN:Si were 0.53 ± 0.04, 0.75 ± 0.05 and 1.28 ± 0.07, respectively (Table 2).

**Table 2:** Chlorophyll *a* (μg L^-1^), inorganic nutrients (ammonium, nitrate, phosphate, silicate), total dissolved and organic nutrients (μM) as well as nutrient ratios over the course of *Dinophysis acuminata* blooms in Northport Bay from 2008–2012.

	2008	2010	2011	2012	All years
Inclusive dates	5/12–7/11	5/11–8/19	4/26–9/2	3/23–8/15	
Chlorophyll *a* (μg L^-1^)	15.3 (2.8)	11.1 (2.6)	20.1 (7.5)	6.8 (1.5)	13.5 (2.6)
Ammonium (μM)	3.38 (1.29)	5.23 (0.93)	2.91 (0.70)	2.78 (0.86)	3.42 (0.46)
Nitrate (μM)	6.97 (1.92)	17.96 (2.65)	10.1 (1.51)	13.2 (2.35)	12.10 (1.13)
Phosphate (μM)	0.84 (0.22)	1.10 (0.23)	1.34 (0.27)	1.47 (0.29)	1.24 (0.14)
Silicate (μM)	22.9 (2.46)	35.0 (3.32)	31.5 (3.95)	41.3 (4.97)	33.7 (2.22)
TDN (μM)	26.7 (3.06)	38.6 (3.28)	32.1 (2.05)	42.8 (2.48)	35.7 (1.48)
DON (μM)	16.7 (1.68)	15.4 (1.98)	19.1 (1.33)	26.8 (2.36)	20.2 (1.10)
TDP (μM)	1.15 (0.16)	2.28 (0.33)	2.18 (0.22)	2.20 (0.38)	2.03 (0.16)
DOP (μM)	0.37 (0.11)	1.18 (0.17)	0.85 (0.09)	0.74 (0.11)	0.80 (0.07)
N:P	12.4 (2.34)	35.1 (7.46)	16.2 (3.48)	20.8 (4.49)	20.6 (2.50)
N:Si	0.41 (0.09)	0.71 (0.09)	0.50 (0.07)	0.51 (0.08)	0.53 (0.04)
DON:DOP	54.7 (9.78)	22.8 (8.96)	35.9 (13.7)	48.2 (6.89)	35.3 (3.85)
DON:Si	0.85 (0.15)	0.50 (0.09)	0.79 (0.09)	0.82 (0.11)	0.75 (0.05)
TDN: TDP	26.0 (2.85)	22.4 (4.31)	16.4 (1.22)	28.7 (4.19)	22.9 (1.73)
TDN:Si	1.25 (0.13)	1.20 (0.13)	1.29 (0.12)	1.33 (0.16)	1.28 (0.07)

Samples were averaged across the respective inclusive dates with standard errors indicated in parentheses.

During the four years DSP toxins were detected (2008, 2010, 2011 and 2012), nitrogen (ammonium, nitrate, TDN, DON) and phosphorus (phosphate, TDP, DOP) concentrations were lower during the peak of the *Dinophysis acuminata* bloom compared to before and after the peak of the bloom, although these decreases were rarely statistically significant (Fig 2 and Table 3). Overall chlorophyll *a* concentrations during the peak of the *D*. *acuminata* bloom compared to before and after the peak of the bloom were highly variable across years (Table 3). Chlorophyll *a* concentrations (34.3 ± 17.4 μg L^−1^) during the large 2011 *D*. *acuminata* bloom were significantly (p<0.01, Mann-Whitney) higher compared to before and after the bloom (11.4 ± 4.9 μg L^−1^; Table 3). Forward stepwise multiple regression analyses were used to determine the dependence of both *Dinophysis acuminata* abundances and cellular toxin content on multiple environmental variables (Table 4). A highly significant model (*R* = 0.87; *p*<0.001) predicted *D*. *acuminata* abundances were positively and strongly dependent on temperature (*p*<0.001), salinity (*p*<0.001), chlorophyll *a* (*p* = 0.002), ammonium (*p* = 0.007), positively, but more weakly dependent on pennate diatoms (*p* = 0.04), and were weakly, negatively dependent on phytoflagellates (*p =* 0.02; Table 4). The best multivariate linear regression model for predicting the cellular toxin content of *D*. *acuminata* during blooms was also highly significant (*R* = 0.60; *p*<0.001) and was positively dependent on ammonium (*p* = 0.002) and negatively dependent on nitrate (*p*<0.001; Table 4).

**Table 3:** Chlorophyll *a*, and mean nutrient concentrations and ratios at the peak of the bloom compared to before and after the peak of the bloom (when cells where present) for Northport Bay 2008–2012.

	2008		2010		2011		2012	
	bloom peak	before and after	bloom peak	before and after	bloom peak	before and after	bloom peak	before and after
Inclusive dates	5/16–5/26	5/12, 5/29–7/11	6/14–6/29	5/11–6/2, 7/21–8/19	6/6–7/11	4/26–6/1, 7/18–9/2	5/29–6/19	3/23–5/22, 6/26–8/15
Chlorophyll *a* (μg L^−1^)	13.2 (3.3)	16.6 (4.0)	13.0 (2.7)	10.2 (3.6)	***34*.*3 (17*.*4)***	***11*.*4 (4*.*9)***	5.2 (4.1)	7.2 (1.6)
Ammonium (μM)	0.7 (0.2)	4.9 (1.8)	4.5 (0.4)	5.5 (1.4)	2.0 (0.8)	3.5 (1.0)	2.6 (1.9)	2.8 (1.0)
Nitrate (μM)	5.2 (1.8)	8.0 (2.9)	10.7 (3.4)	21.2 (3.0)	7.2 (2.4)	12.0 (1.8)	9.5 (2.1)	14.2 (2.9)
Phosphate (μM)	0.4 (0.07)	1.1 (0.3)	1.0 (0.1)	1.1 (0.3)	1.0 (0.3)	1.6 (0.4)	1.1 (0.5)	1.6 (0.3)
Silicate (μM)	23.4 (4.8)	22.7 (3.1)	29.9 (2.6)	37.2 (4.6)	31.0 (6.5)	31.9 (5.2)	38.7 (6.1)	42.0 (6.1)
TDN (μM)	22.2 (2.8)	29.4 (4.4)	34.5 (4.0)	40.5 (4.4)	28.0 (3.1)	34.7 (2.6)	***52*.*3 (4*.*7)***	***40*.*3 (2*.*6)***
DON (μM)	16.3 (1.7)	16.9 (2.6)	19.3 (1.4)	13.7 (2.6)	18.9 (1.7)	19.2 (1.9)	***40*.*3 (5*.*9)***	***23*.*2 (1*.*6)***
TDP (μM)	***0*.*7 (0*.*1)***	***1*.*4 (0*.*2)***	2.6 (0.2)	2.1 (0.5)	2.1 (0.3)	2.3 (0.3)	1.7 (0.5)	2.3 (0.5)
DOP (μM)	0.3 (0.1)	0.6 (0.2)	1.6 (0.2)	1.0 (0.2)	***1*.*1 (0*.*1)***	***0*.*7 (0*.*1)***	0.6 (0.1)	0.8 (0.1)
DIN:DIP	13.9 (4.7)	11.5 (2.8)	15.4 (3.2)	43.8 (9.4)	9.9 (1.7)	20.1 (5.3)	17.4 (7.6)	21.7 (5.4)
DIN:Si	0.27 (0.07)	0.49 (0.12)	0.53 (0.14)	0.78 (0.10)	***0*.*29 (0*.*07)***	***0*.*63 (0*.*10)***	0.37 (0.12)	0.55 (0.09)
DON:DOP	68.9 (15.8)	43.3 (11.0)	12.5 (0.89)	27.4 (12.9)	19.5 (3.3)	24.3 (3.3)	***76*.*3 (18*.*9)***	***40*.*7 (6*.*1)***
DON:Si	0.78 (0.13)	0.90 (0.23)	0.67 (0.12)	0.42 (0.11)	0.82 (0.16)	0.77 (0.11)	1.1 (0.25)	0.74 (0.11)
TDN: TDP	32.5 (5.2)	22.3 (2.7)	13.5 (1.5)	26.4 (5.8)	14.5 (1.8)	17.6 (1.6)	42.1 (14.3)	25.2 (3.6)
TDN:Si	1.0 (0.17)	1.4 (0.18)	1.2 (0.23)	1.2 (0.18)	1.1 (0.15)	1.4 (0.18)	1.5 (0.35)	1.3 (0.18)

Values are means with standard errors in parentheses. 2009 was excluded given the low *Dinophysis* densities. Values that are italicized are those that are significantly different from each other as determined by a t-test.

**Table 4:** Multiple regression of *Dinophysis acuminata* abundances and toxin content per *Dinophysis* cell on environmental variables.

	Significant predictor variables	Standardized coefficient	*p* value	*R*
***Dinophysis acuminata***	Temperature	0.595	<0.001	0.87
**abundances**	Salinity	0.299	<0.001	
	Chlorophyll *a*	0.581	0.002	
	Ammonium	0.240	0.007	
	Phytoflagellates	-0.414	0.019	
	Pennate diatoms	0.164	0.037	
**Total toxin content**	Nitrate	-0.662	<0.001	0.6
	Ammonium	0.578	0.002	

Both models were significant at the *p*<0.001 level.

Consistent with water column temperatures, mean atmospheric temperatures were not significantly different among bloom years (mean ± SD = 17.1 ± 5.4, 16.2 ± 5.6, and 16.4 ± 5.3°C for 2010, 2011 and 2012, respectively). Additionally, there were no statistically significant differences in precipitation or wind intensity among bloom years. Wind direction, however, was significantly different among years (April through June; specifically 2010 vs. 2011, p<0.05; Tukey), with winds blowing from the SW (226 ± 81°) in 2010, S (179 ± 89°) in 2011, and the SW (203 ± 92°) in 2012. The wind direction for April 2011 (S, 184 ± 93°) was significantly (*p*<0.05, Tukey) different than both April 2010 (SW, 242 ± 82°) and 2012 (SW, 246 ± 89°).

### Nutrient amendment experiments

**Fig 3:**
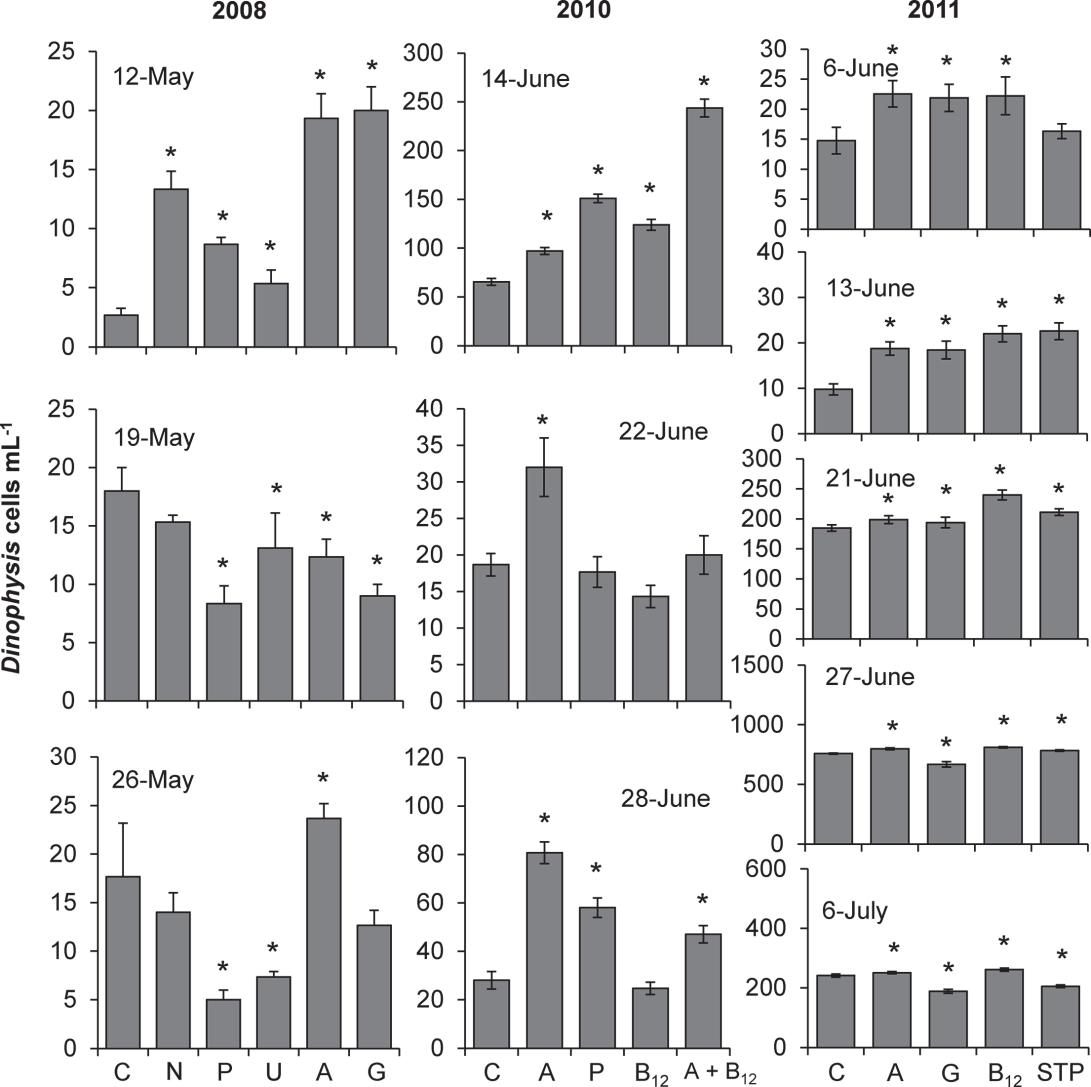
*Dinophysis acuminata* densities (cells mL^-1^) at the end of nutrient amendment experiments conducted during 2008, 2010 and 2011 using water collected from Northport Bay, New York. Bars are means while error bars represent the SD of triplicate bottles. Asterisks indicate treatments that are significantly different compared to the unamended control. C = Control, N = Nitrate, P = Phosphate, U = Urea, A = Ammonium, G = Glutamine, B_12_ = vitamin B_12_, A+ B_12_ = Ammonium + vitamin B_12_, STP = high molecular weight sewage treatment plant organic matter.

**Fig 4:**
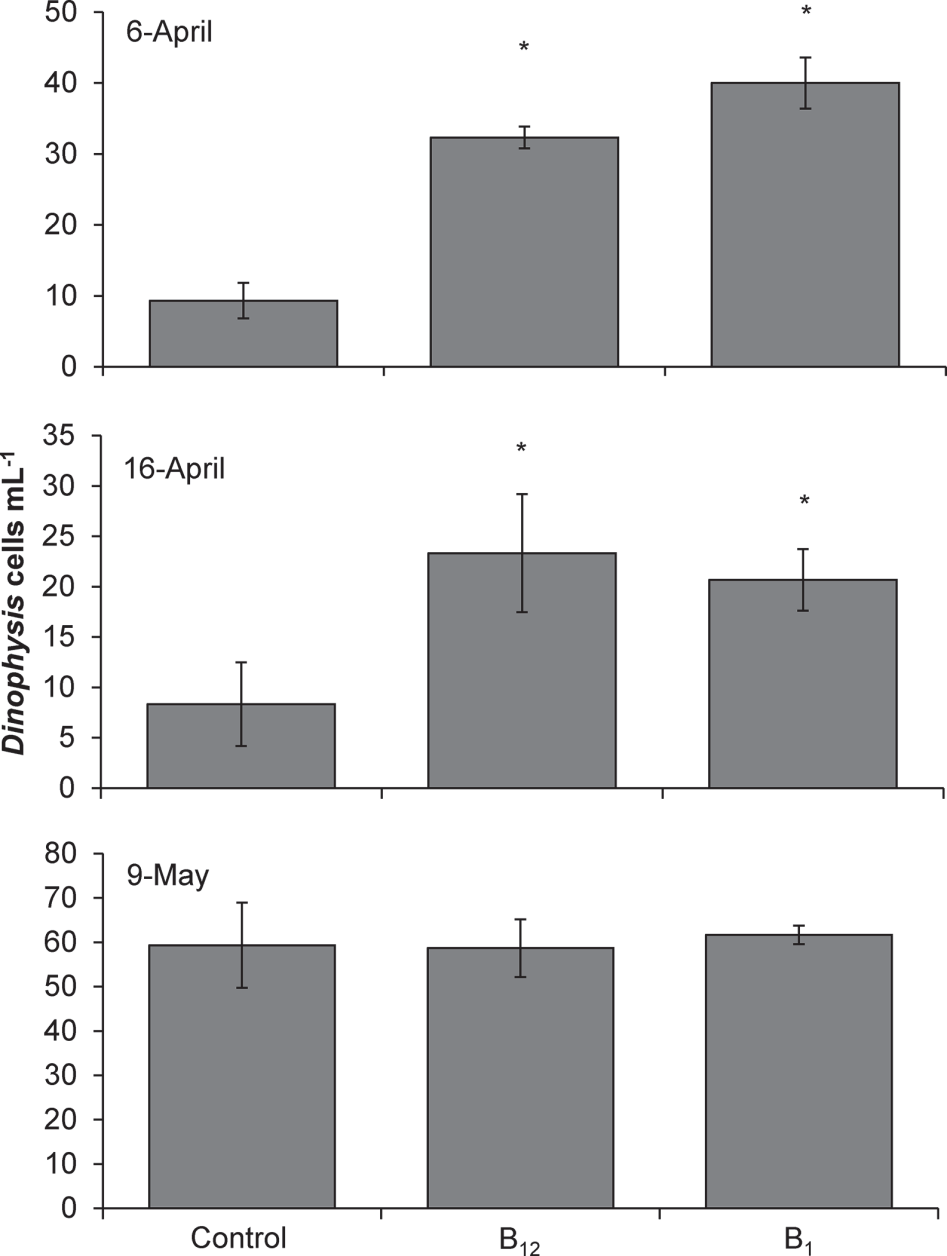
*Dinophysis acuminata* densities (cells mL^-1^) at the end of nutrient amendment experiments conducted during 2011 using water collected from Meetinghouse Creek, New York. Bars are means while error bars represent the SD of triplicate bottles. Asterisks indicate treatments that are significantly different from the unamended control. C = Control, B_12_ = vitamin B_12_ and B_1_ = vitamin B_1_.

The addition of both inorganic and organic nutrients enhanced the abundances of *Dinophysis acuminata* in 13 of 14 experiments performed. In field experiments conducted over three years in Northport Bay, ammonium consistently and significantly increased *Dinophysis* densities (4–625%) compared to the control in nearly every experiment in which that compound was used (10 of 11 experiments; p<0.01 for all; Fig 3). In 6 of 8 experiments performed, the addition of vitamin B_12_ significantly increased *Dinophysis* densities 7–125% compared to the control (p<0.05 for all; Fig 3). High molecular weight organic matter isolated from a sewage treatment plant (HMW STP) significantly increased *Dinophysis* densities up to 130% compared to the control in 3 of 5 experiments with that material (p<0.05 for all; Fig 3). Additions of nitrate, phosphate, urea, or glutamine yielded less consistent results, causing statistically significant increases in *Dinophysis* densities up to 400, 225, 100 and 650%, respectively, in 1 of 3, 3 of 6, 1 of 3, and 4 of 8 experiments performed, respectively (Fig 3). The addition of ammonium and vitamin B_12_ together was examined on 14, 22, and 28 June 2010 and yielded significant increases (up to 270%; p<0.001) in *Dinophysis* densities compared to the control on 14 and 28 June, with only the 14 June yield being significantly greater than either individual addition (Fig 3). In a small fraction of cases *Dinophysis* densities significantly decreased with the addition of nutrients (e.g. 19 and 26 May). Overall phytoplankton growth for both size fractions (whole and >20μm) was positive with the exception of 27-June (2011, whole chlorophyll *a*) and 21-June (2011, >20μm) where net growth rates were negative (S1–S4 Tables). The chlorophyll-specific growth rates significantly increased in response to nitrate and ammonium during most experiments (p<0.05; S1–S4 Tables). During the experiments conducted in Meetinghouse Creek in 2011, the addition of vitamins B_1_ and B_12_ significantly enhanced the abundance of *D*. *acuminata* in two of three experiments, increasing *Dinophysis* densities up to 250% (p<0.05) and 330% (p<0.05), respectively, compared to the control (6 and 16-April; Fig 4).

Spearman’s correlation matrices were generated using the densities of *Dinophysis* and other members of the planktonic community within experimental bottles at the end of experiments performed in 2008, 2010 and 2011. For each year, *Dinophysis* densities were significantly, but inversely, correlated with the densities of autotrophic flagellates (2008: R = −0.46, p<0.05; 2010: R = −0.84, p<0.001; 2011: R = −0.82, p<0.001). In 2008 and 2010, *Dinophysis* densities were also significantly, but inversely, correlated with pennate diatoms (R = −0.54, 2008 and R = −0.34, 2010; p<0.05 for both years) and in 2011, *Dinophysis* densities were significantly, but inversely, correlated with the ciliate, *Mesodinium* (R = −0.28, p<0.01).

**Fig 5:**
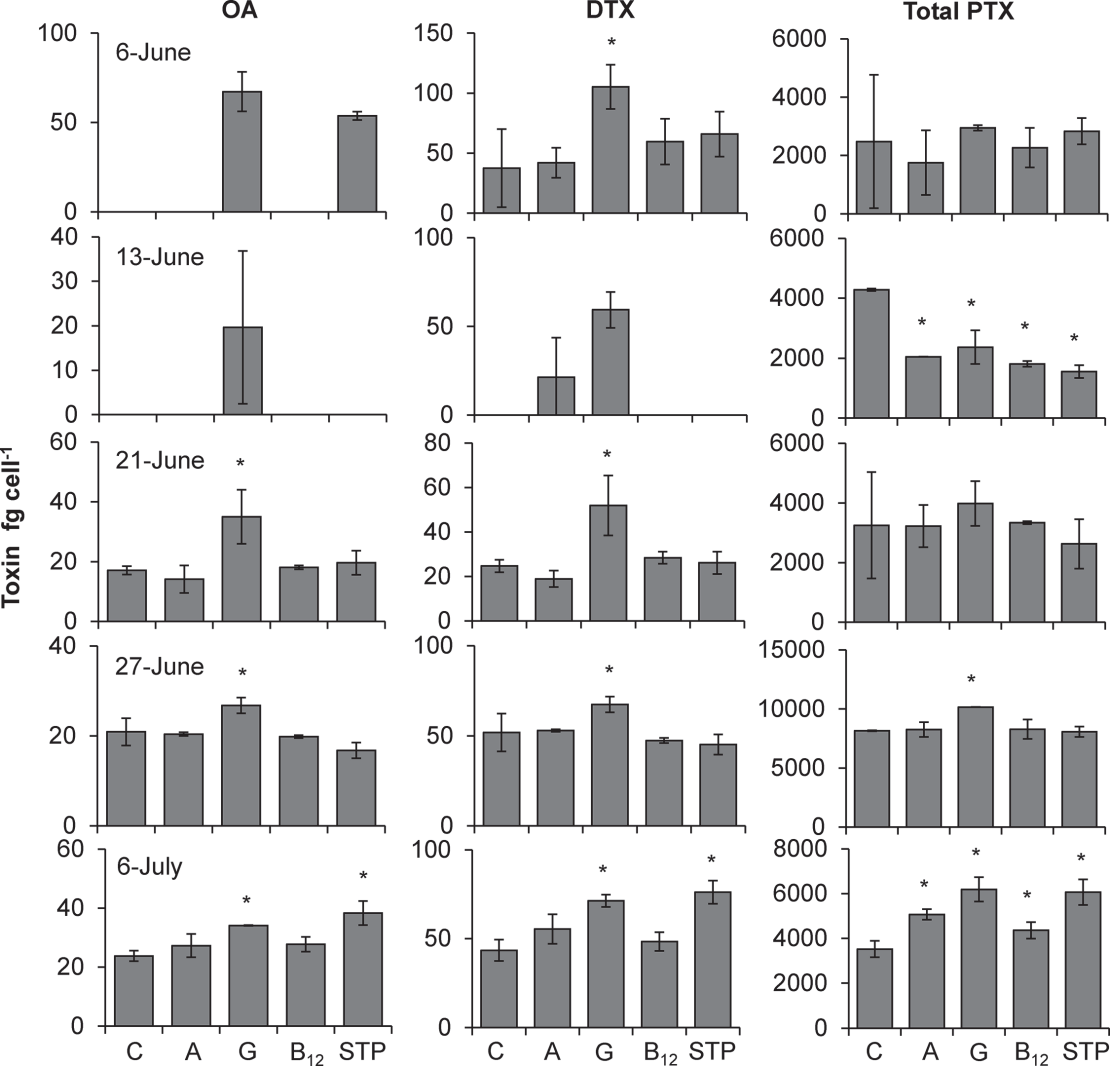
Okadaic acid (OA), dinophysistoxins 1 (DTX1) and total pectenotoxins (total PTX) as fg cell^-1^ for nutrient amendment experiments conducted using Northport Bay bloom water during 2011. Bars are means while error bars represent the SD of triplicate bottles. Asterisks indicate treatments that are significantly different from the unamended control. Abbreviations as in Fig 2.

The addition of inorganic and organic nutrients enhanced the toxicity of *Dinophysis acuminata* in experiments conducted with Northport Bay bloom water during 2011. Glutamine significantly increased toxin content per cell (fg cell^−1^) for okadaic acid (OA; p<0.05, 28–104%), dinophysistoxin-1 (DTX1; p<0.05, 30–181%) and total pectenotoxins (total PTXs; p<0.05, 25–76%) compared to the control in 60%, 80%, and 40% of experiments conducted, respectively (Fig 5). During the experiment conducted on 6-July, the HMW STP addition significantly (p<0.01) enhanced cellular content of OA, DTX1 and total PTX by 61, 76 and 72%, respectively (Fig 5). In a single case (13-June), the addition of all nutrients caused a significant decrease (45–63%; p<0.001) in total PTX content compared to the control (Fig 5). Contrastingly, the addition of all nutrients caused a significant increase (24–76%; p<0.05) in total PTX content during an experiment conducted on 6 July (Fig 5).

## Discussion

Prior to this study, the role of nutrient loading in the occurrence of *Dinophysis* blooms has been largely ignored and links between nutrient loading and DSP events have not been made. This four year field investigation documented two high density (>10^6^ cells L^−1^), toxic *Dinophysis acuminata* blooms in two different New York estuaries, Meetinghouse Creek and Northport Bay. Highly significant multi-variate forward step-wise regression models demonstrated that *D*. *acuminata* abundances and cellular toxin content were both dependent upon concentrations of ammonium during blooms and ammonium consistently enhanced *D*. *acuminata* densities during experiments. Additionally, organic nitrogen compounds such as glutamine, and high molecular weight organic matter from sewage treatment plants increased the cellular toxicity of *D*. *acuminata* during experiments. Finally, densities of *D*. *acuminata* during experiments were inversely correlated with densities of multiple plankton groups suggesting that trophic interactions and/or competition also influenced *D*. *acuminata* abundance. Collectively, this work provides new insight regarding the role of nutrient loading in the occurrence of HABs caused by this mixotrophic alga.

### The effects of nutrients on *Dinophysis* blooms

*Dinophysis acuminata* is a mixotrophic dinoflagellate and, to date, the role of nutrients in the occurrence of DSP events has received little attention [22]. There were multiple lines of evidence generated by this study, however, that demonstrated that nutrient loading promotes *D*. *acuminata* blooms. While nutrient levels within Northport Harbor were high before and after the onset of blooms, during blooms concentrations of nitrate, ammonium, and phosphate were always lower, sometimes by more than five-fold, indicating there was a large nutrient demand during these events by *D*. *acuminata* and co-occurring algae (e.g. *Prorocentrum* spp, *Skeletonema* spp. and euglenoids). Both DIN:DIP and DIN:Si ratios during the peak of the bloom were lower than those found before and after the peak of the bloom, specifically evidencing a strong N demand during these *Dinophysis* blooms. The DIN:DIP only fell below the Redfield ratio (16:1) during the peak of the large (>10^6^) 2011 bloom, perhaps indicating a stronger N demand during that large bloom. During other periods, inorganic N:P ratios were close to Redfield (20.6 ± 2.5), while organic N:P were substantially above it (35.3 ± 3.85) suggesting that although autotrophs had an adequate balance of nutrients, heterotrophs had excess N relative to P. Finally, silicate levels were high and DIN:Si and DON:Si were always below 1 indicating that diatoms were unlikely to have been limited by the supply of silicon during this study [60–62].

During this investigation the initiation of *D*. *acuminata* blooms occurred during late spring and early summer, a period of warming temperatures and increasing salinities associated with lowered groundwater flow [63, 64]. Multi-variate regression models clearly captured this seasonality with *D*. *acuminata* abundances being significantly dependent on high temperatures and high salinity. The seasonality of these events also coincides with seasonally-influenced biogeochemical cycling of nutrients in estuaries. As blooms develop during late spring and early summer, flow rates of nitrate-enriched groundwater [65] become minimal [63, 64] while benthic fluxes of ammonium become maximal [66, 67]. Consistent with this idea, *D*. *acuminata* abundances and toxin content were positively dependent on ammonium concentrations while toxin content was negatively dependent on nitrate suggesting processes that enhance the production of recycled and regenerated ammonium, such as benthic fluxes, may promote *D*. *acuminata* blooms and enhance cellular toxin content. While a forward stepwise multiple regression model demonstrated that *D*. *acuminata* abundances were positively dependent on ammonium concentrations it additionally demonstrated positive and negative dependence on pennate diatoms and phytoflagellates, respectively, suggesting additional modes of nutrition may contribute toward these events, namely mixotrophy and specifically phagotrophy [6, 68]. Anderson, Burkholder (3) linked eutrophication to increases in mixotrophic (phagotrophic) HABs such as *K*. *veneficum* and *Pfiesteria* spp. Burkholder, Glibert (21) hypothesized that most HABs found in eutrophic environments are mixotrophs that are stimulated directly by nutrients, and/or indirectly by elevated nutrients that increase their algal prey. This may be the case for *Dinophysis* blooms observed during this study which occurred in eutrophied environments with significant point and non-point sources of nutrients that directly stimulate the growth of both this alga and others that may serve as prey. The speciation of nitrogen clearly influenced *Dinophysis* blooms in this system with abundances and toxin content being positively dependent on ammonium concentrations and toxin content negatively dependent on nitrate. Nitrate increased chlorophyll *a* levels in all but one experiment in which it was examined but rarely enhanced *Dinophysis* densities, indicating that in addition to being associated with lower cellular toxicity, nitrate loading depressed the relative abundance of *Dinophysis* within the phytoplankton community during blooms.

While relationships between *Dinophysis acuminata* abundances and nutrients in the field were modest, the experimental addition of nutrients to bloom water significantly and consistently (93% of experiments) yielded enhanced *Dinophysis acuminata* densities. Park, Kim (41) has reported *D*. *acuminata* growth rates of 0.95 d^−1^ (a doubling time of 17.5 h) thus confirming that these enhancements (increases up to 650%) are not out of the realm of possibility. Similar to other HABs [3, 11, 14, 15, 18], *D*. *acuminata* densities were significantly enhanced by both inorganic (nitrate, ammonium, phosphate) and organic (glutamine, vitamins B_1_ and B_12_, high molecular weight sewage effluent) nutrients. The mode by which nutrients promote *Dinophysis* population growth may be either direct via nutrients used by *Dinophysis* or indirect via the increases in the abundance of *Dinophysis*’ prey. Significant correlations between *Dinophysis* and its primary prey, *Mesodinium*, have been observed in prior studies of these blooms [32]. Furthermore, during experiments, there were significant inverse correlations with multiple members of the planktonic community including those that can be considered both direct (*Mesodinium*) and indirect (autotrophic flagellates) prey of *Dinophysis*. Given the broad range of nutrients capable of enhancing the abundances of *Dinophysis* during this study, we hypothesize that many of these nutrients are stimulating the growth of various members of the microbial food web which benefit *Dinophysis* as direct (e.g. *Mesodinium*) or indirect (e.g. autotrophic flagellates) prey. The extent to which nutrients are directly or indirectly promoting *Dinophysis* should be assessed in future culture studies of this alga and/or microscopic examination of prey ingestion during blooms.

B-vitamins consistently yielded elevated densities of *Dinophysis* (e.g. 8 of 8 experiments in 2011). A recent survey of 47 strains of harmful algae found that 96% had an absolute requirement for vitamin B_12_ and 80% had an absolute requirement for vitamin B_1_ [14], and further studies have also noted the ability of picomolar levels of vitamin B_12_ to promote the growth of some HABs in coastal systems [69, 70]. Presently, the vitamin requirements of *Dinophysis* spp. and *Mesodinium* spp. are unknown. Tang et al. [14] demonstrated that the cryptophyte, *Rhodomonas salina*, has absolute requirements for vitamin B_1_ and B_12._ Hence, like other nutrients, the ability of vitamins to enhance *Dinophysis* abundances could be direct or indirect if high vitamin levels promote the growth of cryptophytes, such as *Teleaulax* spp., which are consumed by *Mesodinium* spp, and, in turn, are consumed by *Dinophysis*.

The most consistent result (10 out of 11 experiments) in the present study was the statistically significant increase in *Dinophysis acuminata* densities caused by enrichment of ammonium. This finding was consistent with the dependence of *Dinophysis* abundances on ammonium within statistical models constructed during this study and with a study conducted in South Africa which found *D*. *acuminata*–dominated populations had a high affinity for ammonium [44]. Trainer, Moore (33) attributed the greater frequency of *Dinophysis* blooms in Washington, USA, to higher than average flow from the Fraser River during the summers of 2011 and 2012. While the authors linked the enhanced river flow to stratification that promoted those events [33], tributaries are significant point sources of nutrients to estuaries [71] and frontal boundaries, where *Dinophysis* blooms occurred (in WA), are often associated with high nutrient levels [72]. In addition to ammonium, other regenerated forms of N such as glutamine and high molecular weight sewage effluent enhanced *Dinophysis* densities in 4 of 5 experiments in 2011. Several other studies have demonstrated that dinoflagellates typically prefer regenerated N (ammonium) over new N (nitrate) [12, 59, 73, 74]. Collectively, this study demonstrates that regenerated nitrogen and ammonium, in particular, can promote *D*. *acuminata* blooms.

### The effect of nutrients on *Dinophysis* toxicity

There have been very few lab or field studies on the environmental factors that influence *Dinophysis* toxicity. Both Tong et al. [75] and Nielsen et al. [53, 76] demonstrated that light intensity (15–300 μmol photons m^−2^s^−1^) had no effect on toxin quotas for *D*. *acuminata* and *D*. *acuta*. Tong, Kulis (75), however, found that *D*. *acuminata* toxin production and toxin content varied during the growth cycle and toxin production required light. While the effect of light on *Dinophysis* toxicity was not the subject of the present study, during blooms in Northport Bay total cellular toxin quotas were positively dependent on ammonium and negatively dependent upon nitrate concentrations. In experiments conducted with *D*. *acuminata* bloom water, however, the addition of glutamine increased cellular toxin quotas of OA, DTX1 and total PTX in every experiment performed, while the addition of other nutrients had less consistent effects. A study investigating the effects of nutrients on okadaic acid congeners in two *Dinophysis* species came to contrasting conclusions [45]. While *D*. *acuminata* had higher OA concentrations under N-deficient conditions, levels were highest in *D*. *acuta* during nutrient sufficient conditions (+N+P) [45]. An investigation using cultures of the benthic DSP-producing dinoflagellate, *Prorocentrum lima*, found that either N- or P-limitation increased OA concentrations [77]. Consistent with the present study, however, Nagai, Suzuki (55) found that concentrations of PTX2, OA and DTX1 significantly increased in *D*. *acuminata* with the addition of organic substances (obtained from sonicating a *M*. *rubra* culture). Unlike other toxins associated with human health syndromes, such as saxitoxin and domoic acid, okadaic acid, dinophysistoxins and the co-ocurring pectenotoxins are large molecules (molecular weight >800) that are structurally rich in C that do not contain nitrogen. As such, it is possible the organic C from substances like glutamine and high molecular weight sewage treatment plant water may promote toxin production. Biosynthesis studies have demonstrated that okadaic acid is synthesized from acetate, glycine and glycolate [78]. In addition, Souto, Fernandez [79] found that in *P*. *lima* cultures, the addition of various amino acids increased OA per cell compared to control cultures thereby concluding that amino acids are likely involved in toxin biosynthesis. Given the regularity with which glutamine enhanced okadaic acid and other DSP toxin levels during nutrient enrichment experiments, we hypothesize this amino acid is involved in toxin synthesis as a precursor or cofactor in the synthesis reactions. Clearly, more research is needed to fully resolve the effects of nutrients on the toxicity of *Dinophysis*. Regardless, this study clearly demonstrates that glutamine enhances the toxin content of *Dinophysis*.

### The influence of hydrodynamic processes on *Dinophysis* blooms

While this manuscript focuses on the effect of nutrients on *Dinophysis*, a series of studies have demonstrated that *Dinophysis* blooms can be related to various hydrodynamic processes such as cell accumulation forced by wind patterns and stratification [36, 80]. Our multi-variate regression model revealed the association of high *Dinophysis* densities with higher temperature and salinity, likely reflecting the seasonality of these blooms but not water column stratification or density gradients as this system is shallow, well-mixed, and never strongly stratified with respect to salinity or temperature [32]. Consistent with this, among bloom years (2010, 2011 and 2012) there were no significant differences in water column temperature, atmospheric temperature, or precipitation. While there was also no difference in wind intensity among years, wind direction was significantly different and may have influenced blooms [36]. Wind differences were most pronounced during April, two months prior to bloom peaks, with the largest bloom occurring in 2011 when winds were from the south rather than southwest. As such we hypothesize April wind patterns may have influenced the local fluxes of nutrients that supported subsequent blooms rather than the accumulation of cells as south winds would not have promoted an accumulation but rather a dispersal of cells into the bay.

### Conclusions

During this multi-year study, DSP toxins and *Dinophysis* densities were promoted by and significantly correlated with multiple parameters including ammonium. Ammonium consistently enhanced abundances of *Dinophysis* while glutamine consistently enhanced its toxicity. Correlations between *Dinophysis* and multiple plankton groups during experiments suggested that the mechanism by which nutrients promote *Dinophysis* may be direct or indirect via effects on prey items. Controlled culture experiments are needed to further resolve the effects of nutrients on both the phototrophic and mixotrophic growth of *Dinophysis acuminata*. Regardless, this study demonstrates that enhanced nitrogen and organic matter loading in coastal zones are likely to promote both the growth and toxicity of *Dinophysis* blooms.

## Supporting Information

S1 Table Whole chlorophyll *a* (μg L^−1^) concentrations from nutrient amendment experiments conducted during 2008, 2010 and 2011 using water collected from Northport Bay, New York. Values are means (standard deviation) of triplicate bottles.
(DOCX)

S2 Table Mean growth rates (d^−1^) calculated from whole chlorophyll *a* of nutrient amendment experiments conducted during 2008, 2010 and 2011 using water collected from Northport Bay, New York. Values are means (standard deviation) of triplicate bottles. Asterisks indicate treatments that are significantly (*p*<0.05) different compared to the unamended control.
(DOCX)

S3 Table Size fractionated (>20μm) chlorophyll *a* (μg L^−1^) concentrations from nutrient amendment experiments conducted during 2008, 2010 and 2011 using water collected from Northport Bay, New York. Values are means (standard deviation) of triplicate bottles.
(DOCX)

S4 Table Mean growth rates (d^−1^) calculated from >20μm chlorophyll *a* of nutrient amendment experiments conducted during 2008, 2010 and 2011 using water collected from Northport Bay, New York. Values are means (standard deviation) of triplicate bottles. Asterisks indicate treatments that are significantly (*p*<0.05) different compared to the unamended control.
(DOCX)
