# Supplementary material for: Nitrogenous Nutrients Promote the Growth and Toxicity of Dinophysis acuminata during Estuarine Bloom Events
Source: PLoS One. 2015 Apr 20;10(4):e0124148. doi: 10.1371/journal.pone.0124148 (PMC4403995; doi:10.1371/journal.pone.0124148)
Supplement: S1 Table — Values are means (standard deviation) of triplicate bottles. (DOCX) [file pone.0124148.s001.docx]

**Table S1.** Whole chlorophyll *a* (µg L^-1^) concentrations from nutrient amendment experiments conducted during 2008, 2010 and 2011 using water collected from Northport Bay, New York. Values are means (standard deviation) of triplicate bottles.

| **Year** | **Date** | **Initial** | **Control** | **Nitrate** | **Phosphorus** | **Urea** | **Ammonium** | **Glutamine** |
| --- | --- | --- | --- | --- | --- | --- | --- | --- |
| 2008 | 12-May | 23.6 (1.6) | 16.6 (0.5) | 41.5 (2.6) | 15.7 (1.2) | 28.6 (3.0) | 31.0 (2.8) | 22.0 (1.0) |
|  | 19-May | 9.4 (0.7) | 12.7 (0.7) | 15.6 (0.7) | 12.4 (0.2) | 14.0 (0.5) | 17.2 (1.1) | 15.4 (1.1) |
|  | 26-May | 11.4 (0.3) | 11.9 (0.6) | 22.4 (0.7) | 12.0 (0.4) | 20.2 (1.3) | 21.8 (1.1) | 20.7 (1.8) |
|  |  |  |  |  |  |  |  |  |
|  |  | **Initial** | **Control** | **Ammonium** | **Phosphorus** | **B12** | **Ammonium + B12** |  |
| 2010 | 14-Jun | 15.2 (1.0) | 19.6 (2.5) | 28.2 (0.4) | 23.4 (1.2) | 21.5 (0.5) | 30.0 (0.8) |  |
|  | 22-Jun | 17.7 (2.0) | 18.1 (0.9) | 26.0 (1.1) | 16.8 (0.2) | 19.0 (2.0) | 26.8 (1.1) |  |
|  | 28-Jun | 14.1 (0.4) | 15.7 (2.3) | 30.7 (1.9) | 18.9 (1.1) | 15.4 (2.7) | 30.6 (2.3) |  |
|  |  |  |  |  |  |  |  |  |
|  |  | **Initial** | **Control** | **Ammonium** | **Glutamine** | **B12** | **STP** |  |
| 2011 | 6-Jun | 17.2 (0.3) | 25.7 (2.0) | 51.9 (5.6) | 39.4 (2.7) | 38.6 (1.2) | 44.4 (1.9) |  |
|  | 13-Jun | 8.7 (0.2) | 45.6 (3.0) | 68.2 (3.3) | 57.6 (2.0) | 43.7 (1.7) | 55.8 (1.4) |  |
|  | 21-Jun | 21.3 (0.3) | 20.8 (1.3) | 34.9 (0.9) | 22.1 (2.6) | 18.2 (0.1) | 24.8 (0.1) |  |
|  | 27-Jun | 77.7 (3.6) | 19.3 (2.4) | 18.9 (0.01) | 19.5 (0.8) | 18.4 (1.2) | 18.3 (0.5) |  |
|  | 6-Jul | 21.9 (4.1) | 35.0 (2.5) | 49.9 (1.7) | 52.3 (3.7) | 40.5 (5.3) | 46.3 (1.6) |  |
