# Supplementary material for: Nitrogenous Nutrients Promote the Growth and Toxicity of Dinophysis acuminata during Estuarine Bloom Events
Source: PLoS One. 2015 Apr 20;10(4):e0124148. doi: 10.1371/journal.pone.0124148 (PMC4403995; doi:10.1371/journal.pone.0124148)
Supplement: S2 Table — Values are means (standard deviation) of triplicate bottles. Asterisks indicate treatments that are significantly (p<0.05) different compared to the unamended control. (DOCX) [file pone.0124148.s002.docx]

**Table S2.**  Mean growth rates (d^-1^) calculated from whole chlorophyll *a* of nutrient amendment experiments conducted during 2008, 2010 and 2011 using water collected from Northport Bay, New York. Values are means (standard deviation) of triplicate bottles. Asterisks indicate treatments that are significantly (*p*<0.05) different compared to the unamended control.

| **Year** | **Date** | **Control** | **Nitrate** | **Phosphorus** | **Urea** | **Ammonium** | **Glutamine** |
| --- | --- | --- | --- | --- | --- | --- | --- |
| 2008 | 12-May | -0.17 (0.01) | 0.28 (0.03)* | -0.20 (0.04) | 0.10 (0.05)* | 0.14 (0.05)* | -0.03 (0.02)* |
|  | 19-May | 0.16 (0.02) | 0.27 (0.02)* | 0.14 (0.01) | 0.21 (0.02)* | 0.32 (0.03)* | 0.26 (0.04)* |
|  | 26-May | 0.02 (0.02) | 0.37 (0.02)* | 0.03 (0.02) | 0.31 (0.04)* | 0.36 (0.03)* | 0.32 (0.05)* |
|  |  |  |  |  |  |  |  |
|  |  | **Control** | **Ammonium** | **Phosphorus** | **B12** | **Ammonium + B12** |  |
| 2010 | 14-Jun | 0.14 (0.07) | 0.34 (0.01)* | 0.24 (0.03)* | 0.19 (0.01) | 0.37 (0.02)* |  |
|  | 22-Jun | 0.01 (0.02) | 0.20 (0.02)* | -0.03 (0.01) | 0.03 (0.05) | 0.22 (0.02)* |  |
|  | 28-Jun | 0.05 (0.07) | 0.41 (0.03)* | 0.15 (0.03) | 0.04 (0.09) | 0.40 (0.04)* |  |
|  |  |  |  |  |  |  |  |
|  |  | **Control** | **Ammonium** | **Glutamine** | **B12** | **STP** |  |
| 2011 | 6-Jun | 0.22 (0.04) | 0.60 (0.06)* | 0.45 (0.04)* | 0.44 (0.02)* | 0.52 (0.01)* |  |
|  | 13-Jun | 0.90 (0.04) | 1.12 (0.03)* | 1.03 (0.02)* | 0.88 (0.02) | 1.01 (0.01)* |  |
|  | 21-Jun | -0.01 (0.03) | 0.27 (0.01)* | 0.02 (0.06) | -0.09 (0.01)* | 0.09 (0.01)* |  |
|  | 27-Jun | -0.76 (0.06) | -0.77 (0.01) | -0.75 (0.02) | -0.79 (0.04) | -0.79 (0.02) |  |
|  | 6-Jul | 0.26 (0.04) | 0.45 (0.02)* | 0.48 (0.04)* | 0.33 (0.06)* | 0.41 (0.02)* |  |
