# Supplementary material for: Nitrogenous Nutrients Promote the Growth and Toxicity of Dinophysis acuminata during Estuarine Bloom Events
Source: PLoS One. 2015 Apr 20;10(4):e0124148. doi: 10.1371/journal.pone.0124148 (PMC4403995; doi:10.1371/journal.pone.0124148)
Supplement: S3 Table — Values are means (standard deviation) of triplicate bottles. (DOCX) [file pone.0124148.s003.docx]

**Table S3.** Size fractionated (>20µm) chlorophyll *a* (µg L^-1^) concentrations from nutrient amendment experiments conducted during 2008, 2010 and 2011 using water collected from Northport Bay, New York. Values are means (standard deviation) of triplicate bottles.

| **Year** | **Date** | **Initial** | **Control** | **Nitrate** | **Phosphorus** | **Urea** | **Ammonium** | **Glutamine** |
| --- | --- | --- | --- | --- | --- | --- | --- | --- |
| 2008 | 12-May | 10.3 (0.4) | 10.3 (0.8) | 17.7 (3.2) | 8.4 (1.1) | 13.4 (1.4) | 16.9 (1.4) | 15.4 (0.8) |
|  | 19-May | 2.8 (0.5) | 6.6 (0.3) | 7.4 (0.8) | 7.3 (0.6) | 11.2 (1.2) | 10.2 (1.6) | 7.5 (1.5) |
|  | 26-May | 6.5 (1.1) | 6.9 (0.1) | 13.8 (1.4) | 7.5 (0.7) | 12.5 (1.3) | 15.6 (0.8) | 12.7 (1.7) |
|  |  |  |  |  |  |  |  |  |
|  |  | **Initial** | **Control** | **Ammonium** | **Phosphorus** | **B12** | **Ammonium + B12** |  |
| 2010 | 14-Jun | 1.0 (0.2) | 7.2 (0.6) | 11.2 (2.4) | 10.3 (0.3) | 6.2 (0.5) | 12.8 (0.6) |  |
|  | 22-Jun | 1.0 (0.1) | 10.2 (1.0) | 16.8 (2.1) | 10.2 (1.8) | 8.9 (0.8) | 14.0 (2.0) |  |
|  | 28-Jun | 5.3 (0.8) | 8.0 (0.6) | 15.6 (2.0) | 9.4 (0.7) | 8.7 (0.8) | 16.0 (3.7) |  |
|  |  |  |  |  |  |  |  |  |
|  |  | **Initial** | **Control** | **Ammonium** | **Glutamine** | **B12** | **STP** |  |
| 2011 | 6-Jun | 1.3 (0.4) | 4.2 (0.1) | 5.6 (0.9) | 4.4 (0.7) | 3.3 (0.4) | 4.7 (0.4) |  |
|  | 13-Jun | 0.6 (0.1) | 6.8 (0.7) | 8.7 (2.4) | 9.3 (0.3) | 5.7 (0.2) | 7.9 (0.4) |  |
|  | 21-Jun | 8.1 (0.3) | 3.5 (0.04) | 4.6 (0.1) | 2.9 (0.3) | 2.8 (0.1) | 4.0 (0.3) |  |
|  | 27-Jun | 8.9 (1.6) | 9.9 (1.1) | 11.7 (0.4) | 12.8 (1.2) | 11.4 (1.2) | 11.5 (1.0) |  |
|  | 6-Jul | 7.1 (0.7) | 26.3 (2.0) | 33.2 (2.6) | 39.6 (7.8) | 26.8 (4.2) | 31.0 (2.0) |  |
